# Ketamine—50 years in use: from anesthesia to rapid antidepressant effects and neurobiological mechanisms

**DOI:** 10.1007/s43440-021-00232-4

**Published:** 2021-02-20

**Authors:** Samuel Kohtala

**Affiliations:** 1 Laboratory of Neurotherapeutics, Drug Research Program, Division of Pharmacology and Pharmacotherapy, Faculty of Pharmacy, University of Helsinki, P. O. Box 56, 00014 Helsinki, Finland; 2 SleepWell Research Program, Faculty of Medicine, University of Helsinki, Helsinki, Finland; 3 Feil Family Brain and Mind Research Institute, Department of Psychiatry, Weill Cornell Medicine, New York, NY USA

**Keywords:** Ketamine, Subanesthetic, Rapid-acting antidepressant, Depression, Dose, Anesthesia

## Abstract

Over the past 50 years, ketamine has solidified its position in both human and veterinary medicine as an important anesthetic with many uses. More recently, ketamine has been studied and used for several new indications, ranging from chronic pain to drug addiction and post-traumatic stress disorder. The discovery of the rapid-acting antidepressant effects of ketamine has resulted in a surge of interest towards understanding the precise mechanisms driving its effects. Indeed, ketamine may have had the largest impact for advancements in the research and treatment of psychiatric disorders in the past few decades. While intense research efforts have been aimed towards uncovering the molecular targets underlying ketamine’s effects in treating depression, the underlying neurobiological mechanisms remain elusive. These efforts are made more difficult by ketamine’s complex dose-dependent effects on molecular mechanisms, multiple pharmacologically active metabolites, and a mechanism of action associated with the facilitation of synaptic plasticity. This review aims to provide a brief overview of the different uses of ketamine, with an emphasis on examining ketamine’s rapid antidepressant effects spanning molecular, cellular, and network levels. Another focus of the review is to offer a perspective on studies related to the different doses of ketamine used in antidepressant research. Finally, the review discusses some of the latest hypotheses concerning ketamine’s action.

## Introduction

Over the past 50 years, the use of ketamine for anesthesia has become widespread in both human and veterinary medicine. Its safety and short duration and unique mode of action have made ketamine an important drug in emergency medicine and pain management around the world. The study and use of ketamine for the treatment of several new indications, ranging from pain syndromes to drug addiction and psychiatric disorders, is constantly growing. In experimental research, ketamine has unarguably contributed to a better understanding of the glutamatergic system and the development of animal models of schizophrenia [1].

Perhaps the greatest contribution to the legacy of ketamine has been the discovery of its rapid-acting antidepressant effects [2], which has effectively revitalized interest in the development of new antidepressant drugs with a novel mode of action. Indeed, the past decade has seen a rapid increase in the number of research papers associated with ketamine’s antidepressant effects [3]. It can be argued that even beyond its important role in anesthesia, ketamine is a top contender for being the drug with the largest impact for research and treatment of psychiatric disorders in the past several decades.

The ability of ketamine to provide a rapid relief of depressive symptoms, often within hours, has brought it to the forefront of treating severe treatment-resistant depression [4]. Despite its relatively fast pharmacokinetics, the antidepressant effects of ketamine are sustained for up to 1–2 weeks [5]. This rapid onset of ketamine’s action combined with a sustained response lasting far beyond its acute pharmacological effects has puzzled researchers and medical doctors. In contrast, typical antidepressant drugs, such as selective serotonin reuptake inhibitors, often require weeks or even months of continuous medication to elicit their beneficial effects [6]. While the last 2 decades of basic and clinical research have significantly contributed to the understanding of ketamine’s complex profile of effects and its potential for the treatment of psychiatric disorders, the precise neurobiological basis of its antidepressant action remains unclear.

With a vast amount of research aimed at untangling the mysteries of its action, an ever-increasing number of molecular targets and mechanisms have been associated with the antidepressant effects of ketamine [7]. A myriad of novel drug candidates, essentially mimicking certain aspects of ketamine’s action, have risen out of preclinical research. However, to date, no new drug has conformed to the high expectations, with multiple candidates failing in recent clinical trials [8–10]. The shortcomings of animal models of depression are often discussed as a plausible explanation for these failures of translational research, but many other aspects also require further examination. In the rush to understand the molecular complexities of ketamine’s action, both basic and clinical researches have overlooked details related to the administration and dosing time of ketamine [11, 12]. For example, studies directly comparing different dosing parameters and pharmacokinetics between human and rodent studies are still lacking.

With many new ketamine studies published each year, staying up to date becomes a challenge. Thus, the purpose of this review is both to introduce the reader to the various uses of ketamine from anesthesia to pain management and psychiatric use and to provide a thorough overview of the current understanding of ketamine’s effects in treating depression. Herein, the multifaceted profile of ketamine’s effects is reviewed, and studies addressing molecular, cellular, and network mechanisms are introduced. A special emphasis is placed on discussing issues associated with the dosing of ketamine in both basic and clinical research as well as on discussing some of the latest hypotheses related to ketamine’s antidepressant mechanisms.

## An overview of ketamine and its many uses

### What is ketamine?

Ketamine is an anesthetic drug first synthesized in 1962 at the Parke-Davis pharmaceutical company following the discovery of phencyclidine (PCP), another anesthetic belonging to the arylcyclohexylamine chemical class [13]. At that time, several different drugs sharing a similar structure were screened in search of an optimal candidate for further development—preferably one that lacked the unwanted psychotropic effects of PCP. On the basis of the results of these experiments, ketamine became the lead compound for future development. Edward Domino led the first human experiments, which found ketamine to be a safe and short-acting anesthetic. However, ketamine was not entirely devoid of psychotropic effects and was considered a dissociative anesthetic [14].

**Fig. 1:**
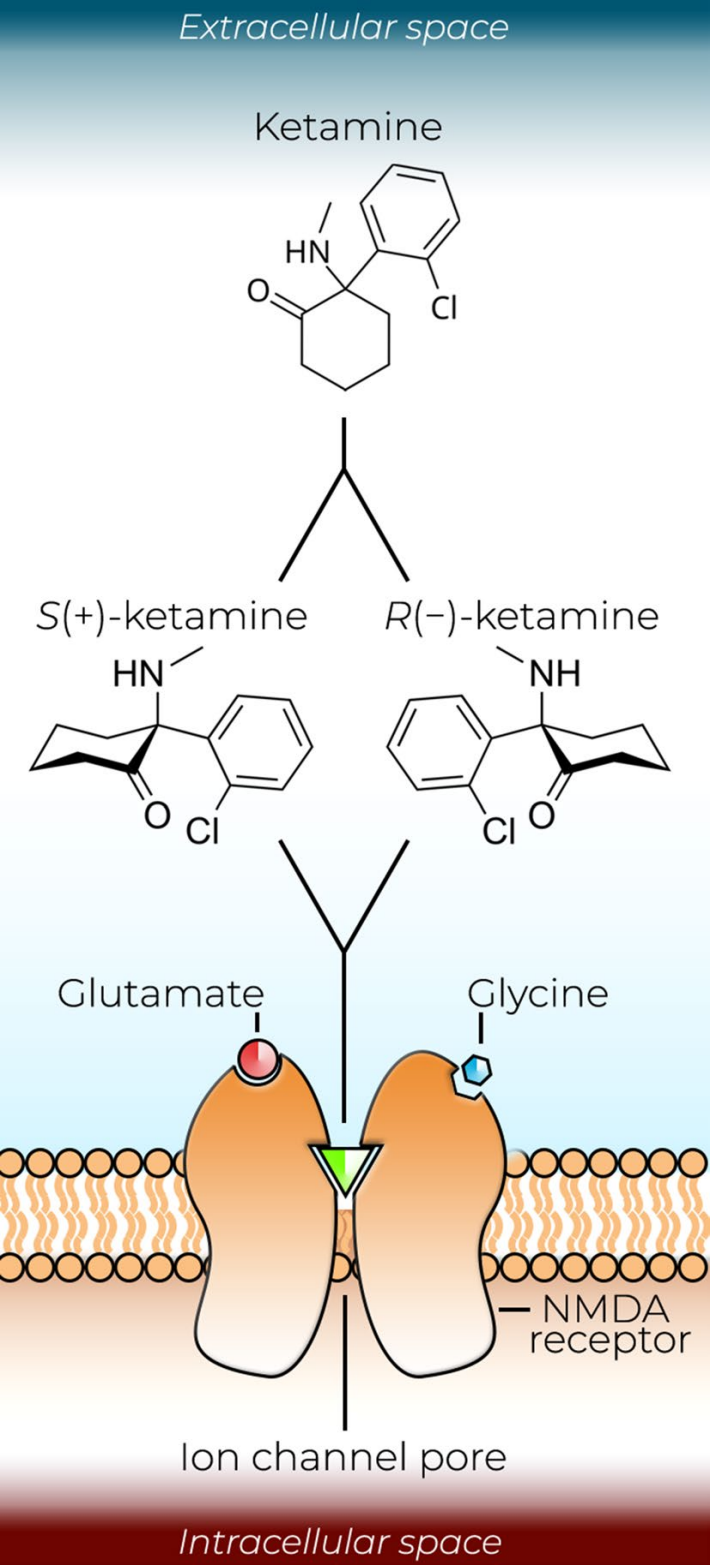
Ketamine acts as a non-competitive blocker *N*-methyl-d-aspartate receptor (NMDAR) ion channels. Racemic ketamine contains two optical stereoisomers: S( +) and R(−)-ketamine. Both isomers act as non-competitive blockers, sharing the ability to enter NMDAR ion channels and to exhibit a trapping block. S-ketamine is often preferred in clinical anesthesia because it has a higher affinity for NMDARs than R-ketamine

Ketamine refers to the mixture of two water-soluble, optical stereoisomers: S( +) and R(−)-ketamine. It is pharmaceutically produced in both racemic and enantiopure preparations. The main mode of ketamine’s pharmacological action is the blockade of *N*-methyl-d-aspartate receptors (NMDARs), ion channels mainly involved in excitatory glutamatergic neurotransmission. Both enantiomers share the ability to block NMDARs but differ slightly in their potency. S-ketamine is often preferred in clinical anesthesia owing to its stronger ability to block NMDARs, whereas R-ketamine has a lower affinity for NMDARs [15]. Ketamine, PCP, and dizocilpine (also known as MK-801)—the classical NMDAR antagonists—are non-competitive inhibitors of NMDARs [16, 17]. By entering the ion channel and then being captured inside the closing pore, they elicit a trapping block (Fig. 1). In contrast, drugs such as memantine act as partial trapping blockers, which only hinder the channel closure but do not entirely prevent it from functioning [18].

Although ketamine exerts its most pronounced effects through the blockade of NMDARs, it has been proposed to also affect many other targets, including dopaminergic, serotonergic, adrenergic, opioidergic, cholinergic, and sigma receptors [1, 15, 19]. Ketamine also acts on serotonin, noradrenaline, and dopamine reuptake transporters (SERT, NET, and DAT respectively) and various ion channels, such as voltage-gated sodium channels (VGSCs) [20] and hyperpolarization-activated cyclic nucleotide (HCN)-gated channels [21].

After administration, ketamine is rapidly distributed in the body and has low plasma protein binding and a short elimination half-life of approximately 2–4 h in humans [22–25]. The rate of metabolism and elimination of ketamine in mice is much faster than that in humans, with a serum half-life of approximately 13 min [26]. The initial metabolite is (R,S)-norketamine (NK), but (2R,6R;2S,6S)-hydroxynorketamine (HNK) and (R,S)-dehydronorketamine (DHNK) are the major circulating metabolites in human plasma [27, 28]. The peak plasma concentration for NK is reached in approximately 1.3 h, and that for DHNK and HNK is reached in 3.8 h [28]. Plasma levels of HNK and DHNK can be measured 24 h after the infusion and may remain detectable for up to 48 h. The initial metabolic reaction—N-demethylation to NK—is mainly catalyzed by liver cytochrome P450 enzymes CYP2B6 and CYP3A4. These steps are followed by hydroxylation to generate HNK and DHNK [29]. In addition, several minor metabolic pathways also exist. For a more thorough take on ketamine’s basic pharmacology, pharmacokinetics, and metabolism, see the review by Zanos et al. [15].

### Dissociation and anesthesia

What makes ketamine unique from most other sedatives and anesthetics is the state of dissociative anesthesia produced by the blockade of NMDARs at high doses. Ketamine does not primarily act through gamma-aminobutyric acid (GABA) receptors like most other anesthetics that possess sedative or hypnotic properties [19]. The dissociative effects of ketamine can be described by the experience of being conscious while being drawn away from sensory perceptions. Higher doses produce a dose-dependent deepening of the dissociative state towards dream-like states of open- and closed-eye visuals and strong perturbations of thought and bodily sensation [30], which may be explained by the unique shifting of intracortical dynamics under the effects of ketamine [31, 32]. A high dose of ketamine results in a state of deep dissociation accompanied by amnesia and loss of consciousness because NMDARs are important components of excitatory neurotransmission, long-term potentiation (LTP), and memory formation. Notably, the psychoactive qualities of ketamine are also sought by recreational users, as evidenced by the surge in using ketamine as a drug of abuse from the 1970s to this day [23].

The electrophysiological effects of ketamine can be measured using electroencephalography (EEG), where ketamine demonstrates different dynamics than many other anesthetics [33]. In nonhuman primates, upon ketamine anesthesia, high beta-gamma electroencephalogram (EEG) oscillations emerge at first and deepen towards slow-delta oscillations [31]. In human volunteers, the loss of behavioral responsiveness has been associated with the onset of EEG slow-wave activity (SWA) [33]. Moreover, high-density EEG studies in human volunteers demonstrate that during ketamine anesthesia, theta, gamma, and delta power increased in frontal and posterior channel clusters, whereas posterior alpha power decreased both under anesthetic and subanesthetic doses [34].

**Fig. 2:**
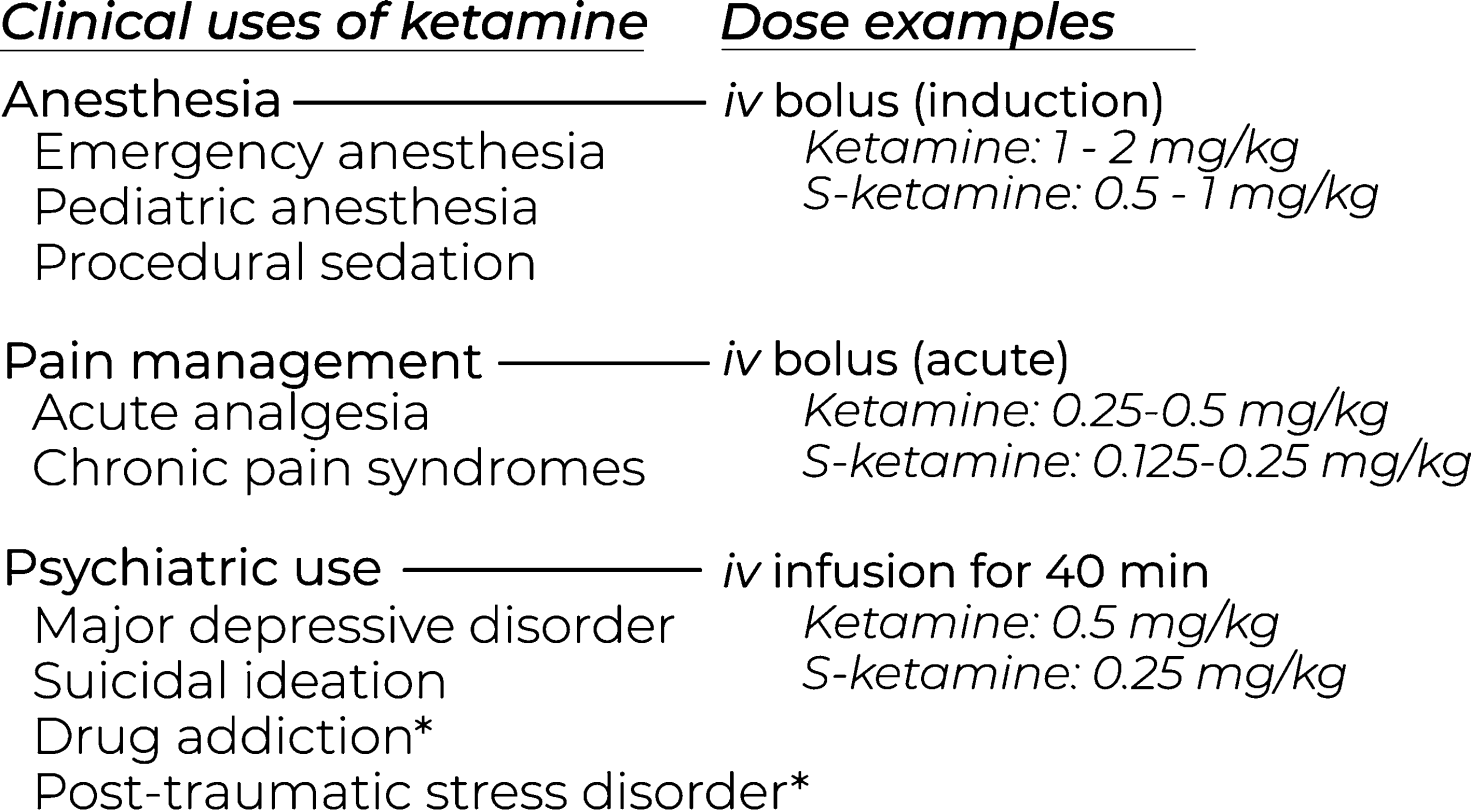
Some of the current and emerging clinical uses of ketamine along with examples of commonly used doses. Clinical applications not yet commonly adopted are followed by an asterix

Owing to its non-GABAergic mechanism of action, ketamine is a safe and effective choice for emergency anesthesia in a prehospital setting. Ketamine offers a relatively wide dosing range, produces a sympathomimetic effect that supports cardiovascular stability along with maintenance of respiratory function, and provides a good level of analgesia comparable to that produced by morphine [35]. During dissociative anesthesia, the basic reflexes are well preserved [36]. For the induction of anesthesia, doses of racemic ketamine are typically in the range of 1–2 mg/kg administered as an intravenous bolus (Fig. 2), which produce a state of dissociative anesthesia within 1–2 min of injection [35]. After induction, a continuous dose of 1–6 mg/kg per h is required to maintain the effect. In contrast, doses of S-ketamine required for the induction of general anesthesia are 0.5–1 mg/kg, followed by a continuous infusion of 0.5–3 mg/kg per h [19]. In veterinary medicine and animal research, ketamine is very commonly used in combination with xylazine, a sedative α_2_ adrenergic receptor agonist [37].

Ketamine can be efficiently administered via multiple routes, including oral, sublingual, intranasal, intramuscular, intraosseous, rectal, and subcutaneous routes, but the highest bioavailability and fastest onset are achieved by intravenous administration [35, 38]. At present, ketamine is used in emergency units for anesthesia and procedural sedation in various patient populations ranging from children to adults. In this context, the wide dosing range, the ability to administer intramuscular doses, and the analgesic properties are particularly useful and make it possible to administer ketamine in field conditions where no anesthesiologist or monitoring equipment is available [39]. However, ketamine is not widely preferred as a sole agent for general anesthesia performed in hospitals owing to its psychotomimetic effects and the potential to produce emergence phenomena, manifesting as profound confusion or hyperexcitation upon waking up from the dissociative state in up to 20% of adults [35].

### Pain management

Ketamine’s analgesic effects, which emerge even at subanesthetic doses, and its good safety profile have prompted its use in the treatment of both acute and chronic pain and in procedural sedation of pediatric patients [40]. The analgesic effects of racemic ketamine become apparent at intravenous doses of 0.25–0.5 mg/kg, which corresponds to 0.125–0.25 mg/kg of S-ketamine [19]. In a prehospital setting, ketamine may produce analgesia comparable to that produced by morphine or fentanyl without as much respiratory depression [41, 42]. For short-term pain relief, low intravenous doses of ketamine provide analgesic effectiveness and safety equivalent to those of intravenous morphine for short-term pain relief [43]. However, ketamine is often used together with other pain medications. For example, a low dose of ketamine combined with a reduced dose of hydromorphone promotes rapid and effective pain relief in emergency department patients suffering from acute pain [44].

A large Cochrane review found that perioperative intravenous ketamine reduces the need for postoperative analgesics and decreases perceived pain intensity [45]. Ketamine alone, however, may not prevent chronic postoperative pain [46–49]. In addition to its use in treating acute pain, ketamine is also used in chronic pain syndromes, where it exerts beneficial effects through the inhibition of NMDARs [50]. Ketamine has been suggested to be effective in treating many different types of chronic pain, such as neuropathic pain [51] and phantom limb pain [52, 53], cancer pain [54], and migraine with prolonged aura [55]. Ketamine may be particularly useful in instances where opioids no longer provide sufficient pain relief because it has been suggested to reduce opioid tolerance and pain hypersensitivity [56, 57]. Moreover, the antidepressant effects of ketamine may be beneficial when treating patients with chronic pain because depression and pain syndromes often co-exist [58].

### Psychiatric use

In the 1970s, the psychoactive side effects of ketamine were desirable in psychiatric investigations where the drug was used as an abreactive agent. A variety of doses more than capable of producing psychotropic experiences were employed in early trials [59, 60] with relatively positive, although anecdotal, outcomes. Moreover, early observations of NMDAR antagonist-induced transient psychotomimetic effects paved the way for the advancement of the idea that glutamatergic neurotransmission was a key component in schizophrenia, and led to the development of ketamine-, PCP-, and MK-801-induced animal models of schizophrenia [61]. More recently, the use of ketamine in treating various psychiatric disorders has regained momentum. Apart from ketamine’s use as a rapid-acting antidepressant drug—which will be more thoroughly discussed in the subsequent section—its use as a novel pharmacotherapeutic intervention for the treatment of post-traumatic stress disorder (PTSD) [62] and substance use disorders [63] has also been explored.

Post-traumatic stress disorder is a condition triggered by the experience of going through a traumatic event [64, 65]. Symptoms may include mental re-experience of the traumatic event and uncontrollable thoughts related to it as well as severe anxiety, arousal, and reactivity. Post-traumatic stress disorder may manifest as negative cognition and mood, and it is often comorbid with depression. Moreover, current pharmacotherapies are often insufficiently effective [66]. Because ketamine is commonly used in emergency anesthesia and analgesia, studies have examined the effects of ketamine administered acutely after physical trauma on the subsequent emergence of PTSD symptoms. On the one hand, two observational studies suggest that ketamine worsens the risk of developing PTSD symptoms [67, 68]; however, questions have been raised about their methodology [62]. On the other hand, a retrospective review of medical records of burn victims suggested reduced prevalence of PTSD among those receiving intraoperative ketamine [69]. However, a subsequent study found no differences [70]. Animal studies of PTSD-like behavior have also shown no changes [71] or slightly detrimental [72] outcomes.

Clinical studies of ketamine in patients suffering from chronic PTSD have been positive. The first randomized controlled study in this context demonstrated that a single subanesthetic dose of *iv* ketamine (0.5 mg/kg) was superior to midazolam (0.045 mg/kg) in rapidly reducing PTSD (and depressive) symptoms within 24 h [73]; however, PTSD symptoms often started to recur 48 h post-infusion, and no significant difference was detected at 1 week post-infusion. Another open-label trial studied repeated iv ketamine infusions (0.5 mg/kg; six infusions over 12 days) and found rapid improvement in both PTSD and depressive symptoms, with the median time to relapse in PTSD remitters being 41 days [74]. Most recently, the first randomized controlled trial of repeated ketamine in PTSD found ketamine to elicit better improvement than the active control group receiving midazolam [75]. Overall, ketamine seems to hold promise in treating PTSD, but more studies are warranted. For a more thorough take on ketamine in the treatment of PTSD, see the review by Feder et al. [62].

Ketamine has been suggested to have beneficial effects in treating drug addiction. Rodent studies have shown that ketamine reduces ethanol consumption in alcohol-preferring rats [76, 77] and reduces morphine-induced place preference [78]. Several clinical studies provide support for the potential of ketamine in the treatment of alcoholism as well as opioid and cocaine addiction and cannabis use disorder [79–85]. In this context, mystical-type experiences mediated by the psychoactive profile of the drug have been suggested to be important [86]. At present, evidence for the use of ketamine in this context is only beginning to emerge, and further research is needed to properly evaluate its effectiveness and safety in treating substance use disorders [63].

## Ketamine as an antidepressant drug

### Rapid onset of action

The seminal study by Berman et al. [2] was the first to demonstrate the rapid-acting antidepressant effects of an intravenous subanesthetic infusion of ketamine in patients suffering from major depressive disorder (MDD). Since then, a number of clinical trials have replicated these results [87–89] and extended the findings to treatment-resistant patients, which has rapidly increased the use of ketamine for the treatment of depression. Other administration routes and doses have also been successfully used, with several trials indicating the antidepressant action of intranasally [90, 91] and orally [92, 93] administered ketamine. However, despite 2 decades of active research, there is still no clear consensus on the dose dependency of ketamine’s effects or the superiority of different administration routes in treating depression.

The factors that separate ketamine from traditional antidepressant drugs are its rapid onset of action and effectiveness in treatment-resistant patients. Ketamine’s antidepressant effects develop within hours of drug administration and may last from a couple of days to approximately 2 weeks following a single dose [5]. The antidepressant effects often peak at 24 h after the infusion [94]. Similar antidepressant effects have also been observed in patients suffering from bipolar depression [95, 96]. These effects of ketamine on depression are remarkably more rapid than those of traditional antidepressants, which may take several weeks or months to develop. Ketamine’s rapid onset of action is particularly useful in treating depressed patients who exhibit suicidality because ketamine also rapidly ameliorates suicidal ideation [97, 98]. Because the antidepressant effects of ketamine are relatively transient, repeated doses are often required to maintain them. However, repeated doses may not produce markedly enhanced antidepressant effects than a single ketamine treatment [99].

### Intravenous, intramuscular, and subcutaneous administration

For the treatment of depression, ketamine is typically infused intravenously over 40 min at a subanesthetic dose of 0.5 mg/kg; the intramuscular and subcutaneous routes are generally less used [38]. The low subanesthetic doses combined with a slow *iv* infusion rate produce only minor psychoactive effects and mitigate the occurrence of dissociative/psychedelic experiences—generally considered unwanted—that become more common towards the higher end of the subanesthetic spectrum. Bolus doses are rarely used owing to poor tolerability. However, an open longitudinal study suggested that a rapid bolus injection of 0.5 mg/kg ketamine also has rapid antidepressant effects with relatively good tolerability [100], whereas a study of S-ketamine (0.25 mg/kg; iv over 10 min) reported that 11.1% of the patients described their experience as very disturbing.

Several small studies have examined the dose-dependent effects of ketamine in depressed patients while also comparing different administration routes. For example, a placebo-controlled cohort-based pilot trial investigated the effects of ketamine on depressive patients using dose titration from 0.1 to 0.5 mg/kg with intravenous, intramuscular, and subcutaneous routes of delivery [101]. The experiment included 15 patients who received ascending doses. Different administration routes produced comparable antidepressant effects, whereas the dose required for an antidepressant response varied between individuals, suggesting that dose titration should be done on an individual basis. In this study, higher doses resulted in greater antidepressant effects and, as expected, more pronounced psychotomimetic effects. Notably, as discussed by the authors, subcutaneous delivery is a promising method for ketamine administration because the plasma levels of ketamine are similar to those observed in intravenous administration and because it is convenient to administer.

Another small dose–response trial compared four different ketamine doses (0.1, 0.2, 0.3, and 0.4 mg/kg) administered to four patients as an iv infusion over 2–5 min in a placebo-controlled double-blind crossover design [102]. The results of this study neatly demonstrate the dose dependency of the psychoactive effects and suggest that antidepressant efficacy of ketamine increases with increasing subanesthetic dose. The dose dependency of ketamine’s antidepressant effects has been further addressed in a systematic review and meta-analysis of nine ketamine trials for the treatment of depression [103]. The authors categorized ketamine doses used in the different studies into low (0.5 mg/kg iv) or very low doses (50 mg intranasal spray, 0.1–0.4 mg/kg iv, and 0.1–0.5 mg/kg iv or sc). Six of these trials used a low dose of ketamine, whereas three used a very low dose. They reported that a low dose of ketamine appeared superior to a very low dose, but a substantial heterogeneity was observed in the clinical response; one-fifth of patients showed remission at 1 week, whereas most others experienced short-lasting benefits. More recently, a double-blind active placebo-controlled trial compared several subanesthetic ketamine doses, but only the higher doses (0.5 mg/kg and 1.0 mg/kg) were found to have clinically meaningful effects [104].

*S*-ketamine has been studied considerably less in terms of the iv dosage range, but 0.25 mg/kg of *S*-ketamine has been shown to be non-inferior to the standard dose (0.5 mg/kg) of racemic ketamine [105]. One trial used 0.2 and 0.4 mg/kg of *S*-ketamine administered intravenously over 40 min and demonstrated robust efficacy [106]. The authors note that the lower dose may allow for higher tolerability while maintaining efficacy.

Taken together, these studies suggest that there is a range of therapeutically active subanesthetic doses of ketamine. For racemic ketamine, doses towards the higher end of the range may be more effective; however, existing studies do not provide conclusive evidence. Large-scale studies comparing the various routes of administration are also lacking, as are studies comparing the effectiveness of different infusion rates. Moreover, it also remains unclear whether there are minimum and maximum effective doses for achieving antidepressant responses and whether patients who do not respond to lower doses would benefit from higher doses—and vice versa.

### Intranasal administration

The majority of studies concerning the antidepressant effects of ketamine have focused on investigating intravenous administration, in which doses are determined by the subject’s weight. In contrast, trials for intranasal ketamine typically use predetermined bolus doses. These intranasal doses of ketamine (and S-ketamine) commonly range between 20 and 100 mg [91, 107–110]. While the bioavailability of intranasal ketamine is estimated to be approximately 25–50% [19, 111], this route of administration is faster to use and more convenient than intravenous infusion. With S-ketamine being approved by the U.S. Food and Drug Administration (FDA) for alleviating symptoms of treatment-resistant depression in 2019, the use of intranasal S-ketamine is likely to increase in the coming years. However, concerns have been expressed over the cost-effectiveness of this intranasal formulation [112].

Intranasal administration of 50 mg of ketamine has shown relatively similar treatment outcomes to those of standard ketamine infusion [91]. Similarly, intranasal S-ketamine doses ranging 28–84 mg have been compared by the pharmaceutical company Janssen in a phase II trial [90]. Remarkably, this study also found that higher doses produced more sustained remission. Similar to racemic ketamine administered via infusion, intranasally administered S-ketamine (84 mg) also reduces suicidality in patients at imminent risk of suicide [113]. The safety and efficacy of intranasal S-ketamine have been studied in several phase III trials; Popova et al. [114] demonstrated a statistically significant effect of intranasal S-ketamine combined with a new oral antidepressant in treatment-resistant depression in comparison with antidepressant/placebo, whereas two other studies failed to find statistically significant differences [108, 109]. In addition, one study addressed intranasal S-ketamine combined with an oral antidepressant for relapse prevention [107]. This study found that the continuation of S-ketamine treatment with an oral antidepressant was superior in delaying relapse than the antidepressant plus placebo.

While intranasal S-ketamine clearly has some beneficial effects in the treatment of depression, the magnitude of these effects, among other things, remains a matter of debate [115–117]. Indeed, a re-analysis of the four phase III studies submitted to the FDA suggests that intranasal S-ketamine may only improve depressive symptoms by reducing 4 points on the Montgomery-Åsberg Depression Rating Scale (MADRS), a scale that ranges from 0 to 60 [118]. At present, it remains unclear how well S-ketamine, or intranasal administration in general, performs when compared with racemic ketamine administered as an intravenous infusion. A recent non-inferiority study comparing intravenous S-ketamine versus racemic ketamine suggests that their antidepressant effects may not be markedly different [105]. Indeed, off-label administration of racemic ketamine remains an attractive option for clinicians for intravenous use because it is readily available, low cost, and easily adaptable to nasal administration via sinus nebulizers.

### Psychoactive effects and the therapeutic context

Most clinical research on ketamine’s antidepressant action has focused on using low subanesthetic doses that minimize the emergence of psychotomimetic side effects. Moreover, the majority of studies have addressed the effectiveness of ketamine from a purely pharmacotherapeutic rather than psychotherapeutic perspective. With the resurgence of psychedelic research and therapy, the issue of the importance of subjective experience and the set and setting has been brought up also in association with ketamine treatment [119]. The underlying idea is that psychedelic therapy can be considered to be a drug-assisted form of psychotherapy rather than pure pharmacotherapy [120]. In this context, psychological factors such as the preceding preparation as well as subsequent integration of the experience are thought to be important for influencing therapeutic outcomes by some researchers. While some preliminary studies of psychedelics support the notion that psychoactive effects and therapeutic context are associated with therapeutic effects [120–122], further research is required to thoroughly address their significance.

Regarding ketamine treatment, these questions have not been studied to any significant extent and remain largely unanswered. However, some clinical studies have suggested that the psychoactive or dissociative effects of ketamine during administration could be associated with producing therapeutic responses. For example, Sos et al. [123] performed a double-blind, crossover, placebo-controlled trial and found a substantial relationship between the antidepressant effects and psychotomimetic effects of ketamine. In this study, the more intense psychotomimetic effects, assessed by the Brief Psychiatric Rating Scale (BPRS), were positively correlated with improved mood ratings on the MADRS 7 days after ketamine infusion. A study by Luckenbaugh et al. [124] also investigated whether psychoactive effects were important for the emergence of antidepressant responses by analyzing 108 treatment-resistant depressive patients from three studies that used a single subanesthetic ketamine dose (0.5 mg/kg iv over 40 min). Two of these were double blind, and the third had an open-label design. The authors found a significant correlation between increased Clinician-Administered Dissociative States Scale (CADSS) score at 40 min and improved Hamilton Depression Rating Scale (HDRS) scores at 230 min and on day 7. Moreover, a more recent study by Niciu et al. [125] reported that dissociative symptoms (also measured by CADSS) were associated with the antidepressant responses after 0.5 mg/kg (iv) ketamine. In contrast, another study found no correlation between CADSS and HDRS responses at any time following the ketamine infusion [126].

Notably, some recent studies have focused on examining the subjective properties of ketamine. A small study—not related to depression—investigating eight cocaine-dependent individuals found that ketamine produced significantly greater acute mystical-type effects (measured by Hood’s Mysticism Scale) than the active control lorazepam [127]. The HMS score, but not the CADSS score, was found to mediate the effect of ketamine on the motivation to quit cocaine on the following day. In another interesting approach, Stocker et al. [128] screened 62 YouTube videos of depressed patients narrating their subjective experience of receiving ketamine and found that 27.4% of the individuals reported an experience of floating that they associated with the amelioration of their depressive symptoms. The connection between treatment response and the floating sensation was subsequently investigated in a double-blind, crossover, placebo-controlled clinical trial, which concluded that the two were not associated [129]. However, identifying clinically usable markers of treatment response and new means to improve the psychological effectiveness of psychoactive pharmacotherapies remains an important avenue for further research.

## Towards a neurobiological understanding of ketamine’s antidepressant effects

### Discovery of the role of NMDARs

Among the first studies to promote the idea of the antidepressant-like action of NMDAR antagonists was the work by Trullas and Skolnick [130] in mice. They demonstrated that the NMDAR antagonists AP-7 and MK-801 reduced the behavioral immobility of animals subjected to the forced swimming test (FST), a classical test to assess antidepressant-like properties. In a subsequent study, Skolnick et al. [131] demonstrated that chronic treatments with traditional antidepressants alter radioligand binding to NMDARs in the cerebral cortex. These results suggested NMDARs as a common target for the action of traditional antidepressants and prompted further investigations into the modulation of glutamatergic neurotransmission in the treatment of depression [132]. Remarkably, the first clinical note of the putative antidepressant effects of d-cycloserine, an NMDAR modulator, was published in 1959 [133], but it took several decades before NMDAR antagonists were considered as potential antidepressant therapeutics.

Since the discovery of ketamine’s antidepressant potential in the late 1990s [2], basic research has focused on identifying the neuronal, molecular, and metabolic targets important for ketamine’s effects. A large number of studies have examined the effects of ketamine in various animal models of depression, with most studies providing strong support for the antidepressant-like action of ketamine in rodents [134–139]. An important research line in the study of ketamine’s effects emerged from the study of chronic stress-induced synaptic alterations and dendritic atrophy [140], also associated with dysfunctional neurotrophic support [141], processes that were found to be positively affected by ketamine [142]. Most importantly, the relative similarity of ketamine’s effects in animal models of depression and in clinical studies provides some basis for the translational significance of these findings.

### Cortical excitation and glutamate

**Fig. 3:**
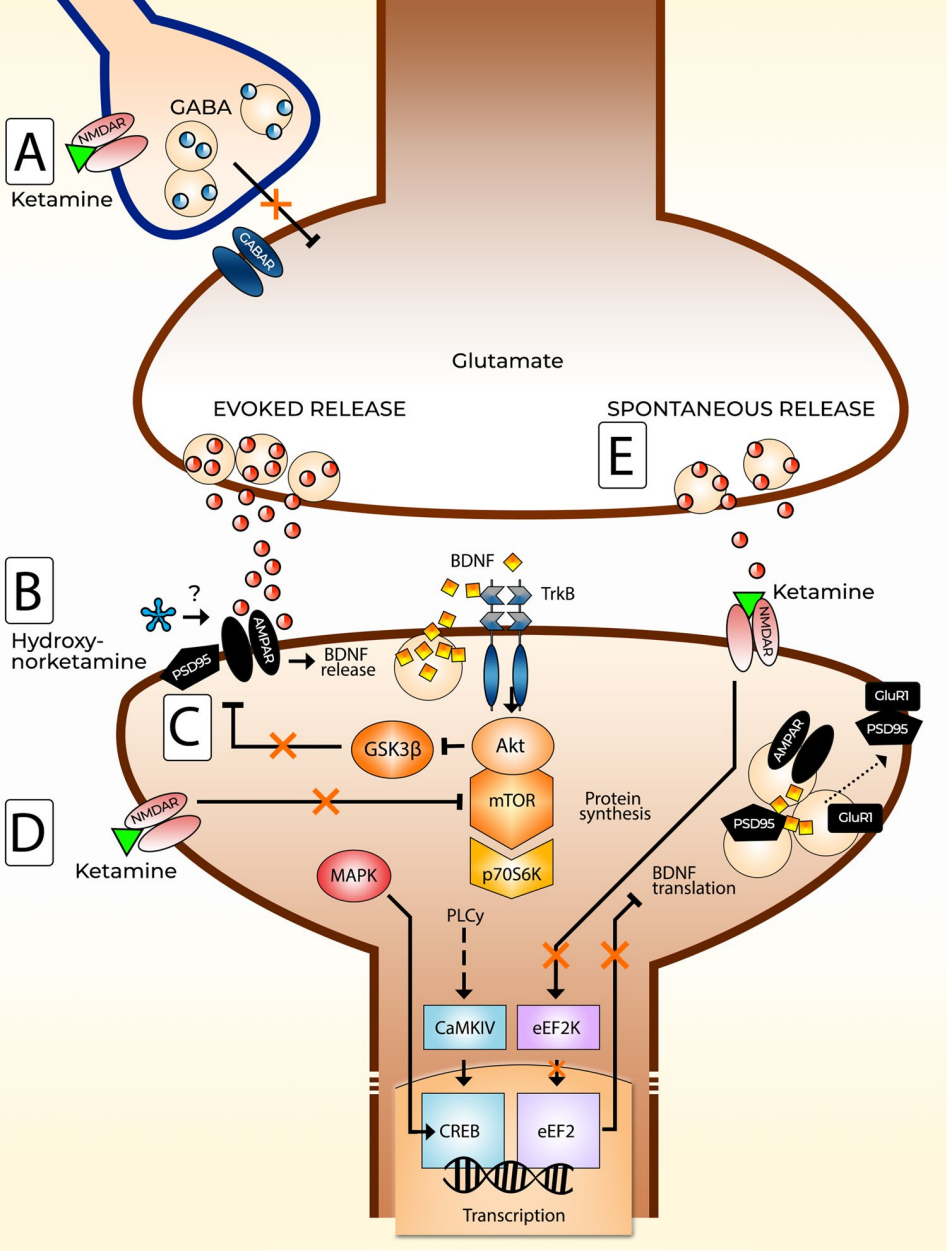
An overview of some of the proposed molecular mechanisms underlying ketamine’s rapid antidepressant action.** a** The disinhibition hypothesis. Ketamine preferentially blocks *N*-methyl-d-aspartate receptors (NMDARs) on gamma-aminobutyric acid (GABA)-ergic inhibitory interneurons, leading to a decrease in the inhibitory tone exerted on excitatory pyramidal neurons. Increased glutamate release acts on postsynaptic α-amino-3-hydroxy-5-methyl-4-isoxazole-propionic acid receptors (AMPARs) and induces cellular effects such as the release of brain-derived neurotrophic factor (BDNF) and activation of its receptor tropomyosin receptor kinase B (TrkB) and the regulation of downstream pathways important for synaptic plasticity and protein synthesis. Downstream effects include activation of mitogen-activated protein kinase (MAPK) and mammalian target of rapamycin (mTOR) and regulation of AMPAR dynamics and scaffolding proteins such as postsynaptic density protein 95 (PSD95). **b** Hydroxynorketamine metabolites may modulate postsynaptic AMPAR signaling, leading to various downstream effects. **c** Inhibition of glycogen synthase kinase 3 β (GSK3β) by ketamine reduces phosphorylation of PSD95, which augments AMPAR signaling by reducing the internalization of AMPAR subunits, among other effects. **d** Ketamine may also block extrasynaptic NMDARs, normally tonically activated by glutamate, and induce mTOR activity. **e** The blockade of spontaneous NMDAR-mediated neurotransmission can have effects that lead to the disinhibition of BDNF translation via eukaryotic elongation factor 2 (eEF2)-dependent mechanisms

The main mechanisms believed to underlie ketamine’s antidepressant effects converge on the increase in cortical excitation and glutamate release and burst, which are thought to trigger subsequent molecular and physiological changes leading to the remediation of depressive symptoms [138]. Because NMDARs are important components of excitatory glutamatergic neurotransmission, one could expect that by blocking these receptors, ketamine treatment would cause cortical inhibition rather than excitation. These paradoxical effects may be explained by the disinhibition hypothesis, which proposes that subanesthetic doses of ketamine preferentially inhibit NMDARs present in GABAergic interneurons (Fig. 3a). This decreases the inhibition of excitatory pyramidal neurons and increases glutamate release and burst [132, 143–145], which continue to activate neurotrophic signaling mechanisms leading to the amelioration of chronic stress-induced synaptic deficits [146]. Two recently published animal studies strongly support the disinhibition hypothesis [147, 148] as the mechanism for subanesthetic ketamine-induced neuronal activation.

Other proposed molecular hypotheses of ketamine’s action include the direct antagonism of extrasynaptic NMDARs on pyramidal neurons, which disrupts the tonic activation of NMDARs by ambient glutamate and results in homeostatic synaptic plasticity and a compensatory increase in excitatory drive in the prefrontal cortex (Fig. 3d) [145]. These changes are thought to be mediated by the blockade of extrasynaptic NMDARs containing GluN2B subunits [149]. Furthermore, ketamine has been suggested to inhibit NMDAR-mediated spontaneous neurotransmission (Fig. 3e) [150] and to trigger an increase in the translation of proteins such as the brain-derived neurotrophic factor (BDNF), a crucial mediator of synaptic plasticity, by reducing the phosphorylation of eukaryotic elongation factor 2 (eEF2), thus leading to antidepressant-like effects [150–152]. In contrast, several recent studies suggest that ketamine’s effects are mediated through its HNK metabolites independently of NMDAR inhibition (Fig. 3b) [134]. Altogether, these hypotheses are not mutually exclusive and may together explain the molecular changes observed after ketamine administration.

Regardless of the chosen hypothetical framework, studies have demonstrated that ketamine possesses excitatory effects that are highly dose dependent. In rats, anesthetic doses of 200 mg/kg (ip) decreased acute glutamate activity measured by microdialysis, whereas lower doses (10, 20, and 30 mg/kg) increased glutamate outflow in the prefrontal cortex [144]. In another study, subanesthetic ketamine provoked transient changes in glutamate cycling in the medial prefrontal cortex of rats [153]. The glutamate surge following ketamine administration may produce its rapid-acting antidepressant effects through the modulation of α-amino-3-hydroxy-5-methyl-4-isoxazole-propionic acid receptors (AMPARs) because the blockade of these receptors abolishes ketamine’s antidepressant-like behavioral responses in mice and rats [154–156]. Moreover, positive allosteric AMPAR modulators produce antidepressant-like behavioral effects in rodents (Knapp et al., 2002; Li et al., 2001) and upregulate BDNF synthesis [159, 160].

### Intracellular signaling pathways

Based on rodent studies, ketamine-induced activation of AMPARs is thought to result in the modulation of a subset of molecular pathways involved in synaptic plasticity, which lead to antidepressant-like effects. For example, ketamine induces the rapid translation and release of BDNF [150, 161], resulting in the activation and phosphorylation of the BDNF receptor, tropomyosin receptor kinase B (TrkB) [150, 162]. BDNF-TrkB signaling constitutes a major component of synaptic plasticity regulation [163, 164], which is crucial for counteracting the negative synaptic effects of stress, restoring altered network activity and producing antidepressant-like effects [142, 146, 165–168]. Indeed, the antidepressant-like effects of subanesthetic ketamine and its HNK metabolites are diminished in BDNF^met66met^ knock-in mice that exhibit compromised activity-dependent BDNF release [169, 170]. The rapid onset of ketamine’s antidepressant effects is hypothesized to be associated with the rapid activity-dependent release of BDNF. However, somewhat unexpectedly, our recent studies demonstrate that the activation of TrkB and associated signaling pathways is more prominent with high, sedative-anesthetic doses of ketamine than with subanesthetic doses of ketamine [171] and sedative doses of non-excitatory drugs such as medetomidine [172].

Other downstream signaling pathways and targets considered to be important in ketamine’s effects are glycogen synthase kinase 3 beta (GSK3β) [135, 150], extracellular signal-regulated kinase 1/2 [ERK1/2, also known as p44/42-mitogen-activated protein kinase (p44/42-MAPK)] [173], and mammalian target of rapamycin (mTOR) [136] along with its effector p70-S6 kinase (p70-S6K) [174]. In mice, the inhibition of GSK3β by ketamine through the phosphorylation of the serine 9 residue has been suggested to be necessary for the rapid antidepressant-like effects observed [135], which may lead to the augmentation of AMPAR-mediated signaling by diminished internalization of GluA1 subunits (Fig. 3c) [175]. Studies investigating ketamine together with co-administered lithium—an unspecific GSK3 inhibitor—found additive antidepressant-like effects in rodents [176]; however, lithium did not facilitate or prolong the effects of ketamine in depressed patients [177]. Moreover, it is important to remember that GSK3β is a promiscuous kinase involved in various cellular processes [178] and the serine 9 phosphorylation is also regulated by several anesthetic drugs other than ketamine [171, 172, 179, 180].

Increased glutamate activity under the effects of subanesthetic ketamine leads to upregulation of p44/42-MAPK activation, whereas higher anesthetic doses reduce MAPK phosphorylation [136, 171]. Increased MAPK signaling is correlated with the antidepressant effects of ketamine in rodents [173], which could be associated with its role in regulating AMPAR trafficking and synaptic potentiation [181]. Indeed, studies have demonstrated that the acute blockade of MAPK signaling results in a depressive phenotype in rodents, and that BDNF ± mice are more sensitive to develop a depressive phenotype when administered a low dose of MAPK kinase inhibitor [182]. Moreover, increased expression of mitogen-activated protein kinase phosphatase 1 (MKP1), a negative regulator of MAPK, results in the emergence of depressive behavior in rodents, whereas mice lacking MKP1 are resilient to stress [183].

Ultimately, activity-induced signaling cascades such as MAPK mediate their effects to the nucleus, where the activity of transcription factors is regulated [184]. Subsequent changes in the expression of immediate early genes (IEGs), particularly those encoding proteins involved in synaptic transmission and function, could be involved in the emergence of antidepressant effects. Here, ketamine-induced expression of IEGs such as *Homer1* [185–189] could be particularly important. This notion is supported by a recent study, where the administration of cell-permeable TAT-Homer1a essentially recapitulated the behavioral responses induced by ketamine in rodents [190].

Finally, the molecular determinants of ketamine’s action are thought to converge on the mTOR complex, which is involved in cellular protein synthesis and metabolism. As demonstrated by Li et al. (2010), subanesthetic doses of ketamine increase levels of synaptic protein and formation of dendritic spines in the prefrontal cortex of rats. Ketamine acutely induces the phosphorylation of mTOR, p70-S6K, eukaryotic translation initiation factor 4E-binding protein 1, p44/42-MAPK, and Akt, which are believed to influence the subsequently upregulated expression of synaptic proteins such as Arc, synapsin I, postsynaptic density protein 95 (PSD95), and GluR1 [136]. Notably, the effects of ketamine on synaptogenesis and behavior are reversed with the blockade of mTOR activity by the intracerebroventricular administration of rapamycin [136]. Following these pivotal findings, several other animal studies have investigated the role of mTOR in the therapeutic effects of ketamine, with promising results [137, 191, 192]. In particular, mTOR has been suggested to be involved in the sustained, rather than acute, antidepressant effects following ketamine administration [137]. However, understanding the function of the mTOR complex in ketamine’s antidepressant effects is complicated by a recent clinical trial, which found that mTOR inhibitor rapamycin increases the duration of ketamine’s antidepressant effects rather than diminishing them [193].

### Cellular-level findings in animal models

Several studies have identified the actions of ketamine on the infralimbic prefrontal cortex (IL-PFC). The inactivation of this region using muscimol blocked the antidepressant effects of systemic ketamine, whereas microinfusion of ketamine into the IL-PFC essentially recapitulated them [194]. Optogenetic targeting and stimulation of the IL-PFC was also found to produce both rapid and sustained antidepressant effects and to increase the number and function of dendritic spines. Recent rodent studies have further elaborated the cellular targets underlying these effects by examining the role of pyramidal neurons expressing dopamine receptor D1 and D2 (Drd1 and Drd2, respectively) [195]. Optogenetic activation of Drd1 pyramidal cells in the medial PFC resulted in rapid and sustained antidepressant effects, whereas stimulation of Drd2 neurons was found to be ineffective.

In rodents, the ketamine-induced amelioration of depressive-like behavior is particularly associated with acute changes in prefrontal circuit function, which is followed by increased dendritic spine formation [137]. The increase in or restoration of dendritic spines is not required for exerting ketamine’s immediate effects but are instead critical for sustaining the antidepressant effect over time. Moreover, interference with ketamine-induced prefrontal spine formation blocked effects on motivated escape behavior but had no influence on sucrose preference, indicating that spinogenesis in other brain areas may support other behaviors [137].

Ketamine also decreases the activation and burst firing of neurons in the lateral habenula (LHb), effects that were associated with acute antidepressant effects in congenitally helpless rats [196]. The local blockade of either NMDARs or low-voltage-sensitive T-type calcium channels in the LHb was found to be sufficient for the induction of rapid antidepressant effects. Essentially, these findings suggest that ketamine’s mood elevating effects result from the blockade of NMDAR-dependent LHb bursts, which disinhibits downstream monoaminergic reward centers [196].

### Electrophysiological and metabolic measures

In terms of more general mechanisms, subanesthetic doses of ketamine increase metabolic activity in the prefrontal cortex in healthy volunteers, as measured by [^18^F]-fluorodeoxyglucose positron emission tomography (PET) [197]. These findings correspond well to other findings suggesting increased glutamate neurotransmission in the prefrontal cortex of healthy and depressed subjects receiving ketamine [198, 199]. The disinhibition hypothesis, which entails a decrease in the activity of GABAergic interneurons and disinhibition of excitatory pyramidal neurons, also provides a putative mechanism for the commonly observed increase in high-frequency gamma oscillations on cortical EEG following ketamine administration [172, 200–202]. Notably, Nugent et al. [202] found that large increases in gamma power were associated with better antidepressant outcomes in subjects with MDD with lower baseline gamma, whereas this relationship was inverse in subjects with MDD with higher baseline gamma. Ketamine also enhanced gamma responses to somatosensory stimuli at 230 min and day 1 in ketamine responders when compared with non-responders, suggesting that increases in synaptic strength coincide with antidepressant effects [203, 204]. A 7 T ^1^H-magnetic resonance spectroscopy study showed no difference in glutamate levels of the pregenual cingulate cortex 24 h post ketamine administration, suggesting that glutamate levels are not altered in the long term [205]. These studies, coupled with abundant molecular evidence, support the notion that subanesthetic ketamine acutely increases cortical excitation and glutamate activity, which drive subsequent synaptic alterations.

In rats, lower doses (60 mg/kg) of ketamine promote behavioral arousal along with theta-range EEG activity and induction of Fos immunoreactivity in the arousal system [206]. In contrast, rats treated with higher doses (150 mg/kg) of ketamine are briefly sedated, but signs of hyperarousal emerge once the acute effects of ketamine subside. Notably, the arousal-promoting effects of subanesthetic doses of ketamine are followed by the increase of delta frequency power during subsequent sleep [207]. Similar intriguing findings were also observed in human studies, in which increased sleep SWA the night following subanesthetic ketamine administration was associated with the therapeutic efficacy of the treatment [208]. The facilitation of SWA is also evident after sleep deprivation, electroconvulsive therapy, repetitive transcranial magnetic stimulation (rTMS), and nitrous oxide administration in humans—all treatments with rapid antidepressant potential [172, 209–211]. These observations suggest that the facilitation of SWA is a homeostatic response to the preceding cortical activation elicited by ketamine and other treatments potentially sharing rapid antidepressant effects [12, 209].

### Clinical neuroimaging

The neuroimaging literature related to ketamine’s antidepressant effects has identified convergent brain regions of interest in the prefrontal cortex, including specific cortical areas such as the subgenual anterior cingulate cortex (sgACC) and the posterior cingulate cortex (PCC) as well as the hippocampus (for a thorough review, see Ionescu et al. [212]). The sgACC, in particular, has been a focus of numerous studies because earlier studies have found depressed patients to exhibit overactivity of the sgACC, which is suggested to normalize upon recovery after treatment [213–216]. A recent study in nonhuman primates demonstrated that ketamine is indeed able to reverse the depressive-like impairments and metabolic changes produced by the overactivation of the sgACC [217]. The hyperactivity of the sgACC has also been observed during task performance in depressed patients, where ketamine may act to normalize sgACC hyperactivity to positive incentives [218]. However, many findings related to the activity of the sgACC remain inconclusive. For example, PET studies have reported no change [219] or increased metabolism in the sgACC in response to ketamine in depressed patients [220]. One study compared ketamine and lanicemine, a promising putative rapid-acting antidepressant at the time, and found them to increase sgACC activation in depressed patients [221]. Since then, lanicemine development has been abandoned after a failure to meet trial endpoints [10]. Nevertheless, another trial found ketamine, but not lanicemine, to increase prefrontal global connectivity [222], hinting at a possible difference between effective and non-effective NMDAR antagonists. This effect of ketamine on connectivity has also been observed in other trials [223, 224] and hypothesized to represent the effects of ketamine on the induction of glutamate neurotransmission.

One focus of functional imaging studies is related to the activity and connectivity of the default-mode network (DMN), which correlates with the severity of depression and rumination [225–227]. A recent double-blind, placebo-controlled, crossover study demonstrated that the connectivity between insula and the DMN was normalized when measured 2 days after ketamine treatment [228]. This change was reversed after 10 days, which corresponds well with the duration of ketamine’s antidepressant effects. Moreover, a study examining ketamine’s effects on fMRI activity during an emotional processing task showed that participants with MDD exhibited greater brain activity than healthy controls after placebo infusion, whereas ketamine reduced brain activity to a level similar to that in placebo-treated healthy controls [229]. Reduction in activity was observed in large regions throughout the brain, including areas related to the DMN. Several studies by the same group support the notion of ketamine normalizing patterns of brain activity in MDD patients [202, 228, 230].

## Emerging hypotheses of ketamine’s action

Understanding the precise mechanistic basis of ketamine’s antidepressant effects has been the target of hundreds of studies in the past decades. These studies have verified the ability of subanesthetic ketamine doses to boost glutamatergic firing and to increase AMPAR function, which continue to be among key areas of interest [7, 138]. For many researchers, the prevailing view is that ketamine has almost a unique ability to regulate a chain of molecular events connected with the facilitation of synaptic plasticity, ultimately steering functional activity towards the amelioration of depressive symptoms. This view has prompted the targeting of individual effectors within complex signaling pathways with the deterministic aim of isolating the root of antidepressant action. These efforts have not been entirely in vain because several studies have demonstrated the antidepressant-like behavioral actions of BDNF [231] as well as the direct activation of TrkB [232] and mTOR [233] in rodents. However, these perspectives may fail to appreciate the full complexity of the molecular signaling events associated with neuronal plasticity, where single effectors are part of a much larger web of activity-dependent molecular interactions. Most importantly, clinical evidence strongly connecting any of the proposed molecular targets to the amelioration of depressive symptoms is currently lacking.

**Fig. 4:**
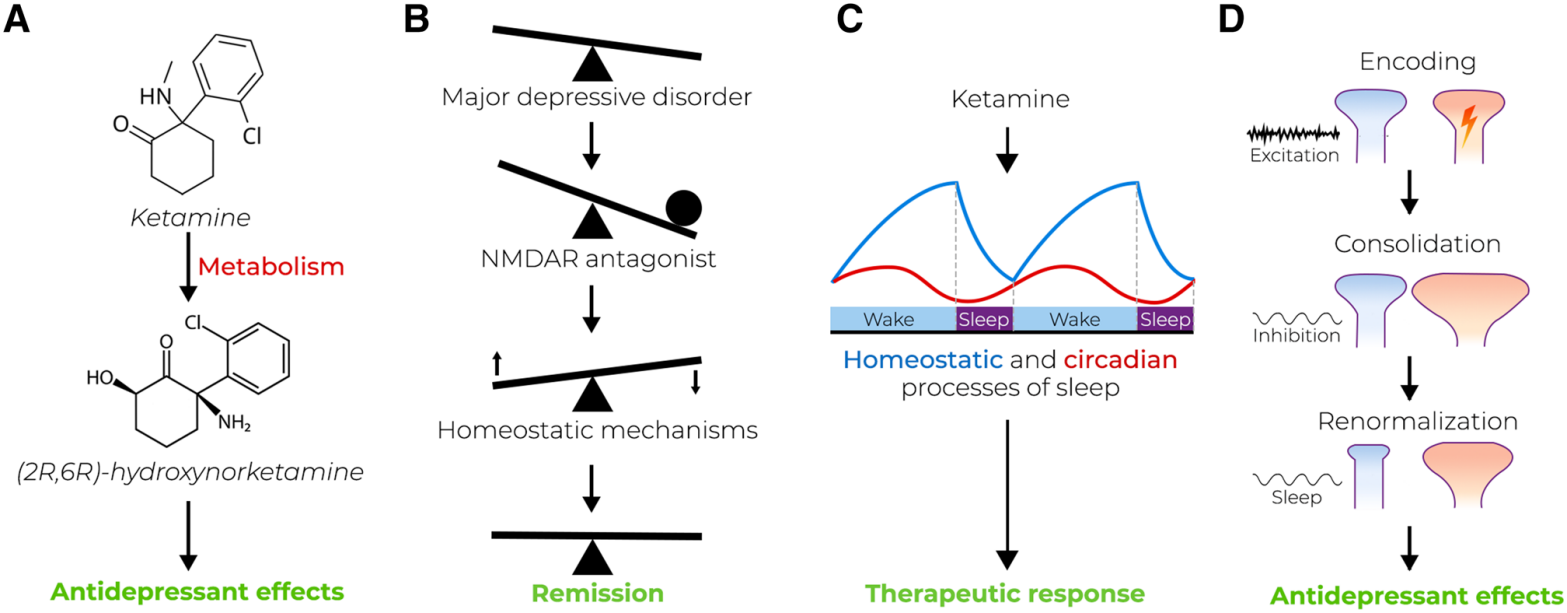
Emerging hypotheses of ketamine’s action. **a** The metabolism of ketamine to its hydroxynorketamine metabolites, and particularly *(2R,6R)*-hydroxynorketamine, is responsible for mediating the rapid antidepressant effects. **b** Ketamine disrupts neuronal homeostasis, which triggers self-correcting mechanisms. **c** Ketamine regulates both circadian and homeostatic processes of sleep to elicit antidepressant effects. **d** The hypothesis of encoding, consolidation and renormalization in depression (ENCORE-D) proposes that ketamine alters neural encoding of information, the consolidation of synaptic change, and the renormalization of synaptic strength during sleep to elicit acute and sustained antidepressant effects

The remarkably rapid yet relatively sustained antidepressant effects of ketamine—often emerging within hours and lasting for up to a week—are a challenging problem to understand. Notably, the recent discovery of the antidepressant-like effects of ketamine’s HNK metabolites have led to a hypothesis in which the gradual buildup of these metabolites, (2R,6R)-hydroxynorketamine in particular, has been suggested to explain ketamine’s antidepressant potency [134] (Fig. 4a). The HNK metabolites differ from the pharmacological effects of ketamine and challenge the notion of ketamine’s effects being dependent on NMDAR inhibition. Antidepressant-relevant concentrations of (2R,6R)-hydroxynorketamine do not block NMDAR function but still result in antidepressant-like responses in rodents [234]. This hypothesis, however, remains controversial in light of more recent preclinical [171, 235, 236] and clinical data [237, 238]. More specifically, recent studies evaluating the plasma levels of these metabolites after ketamine treatment reported contradictory observations—higher levels of 2R,6R-HNK were associated with less clinical improvement [237, 238]. These metabolites remain the topic of ongoing research and debate, while patient studies are still underway.

Several new hypotheses emerged in the recent years explore the neurobiological phenomena underlying antidepressant effects instead of solely crediting ketamine, its metabolites, or any other specific molecular entity for the amelioration of depressive symptoms. It can be argued that because ketamine is rapidly metabolized, its lasting antidepressant effects must be the result of neurobiological adaptations triggered during the brief period of its acute pharmacological action. On the basis of the noteworthy findings of rapid antidepressant-like effects produced by NMDAR enhancers in rodents and those showing the antidepressant-like effects of the negative allosteric GABA-A receptor modulator MRK-015, Workman et al. [239] proposed that rapid-acting antidepressants engage mechanisms of homeostatic plasticity to treat depression (Fig. 4b). In particular, they highlight the ability of these treatments to either strongly increase or decrease neuronal activity, which causes neurons and neuronal circuits to homeostatically self-correct. In this context, ketamine and other putative treatments may act as disruptors of the homeostatic balance, triggering the activation of self-correcting homeostatic mechanisms that are otherwise insufficiently activated in MDD. Importantly, their work also discusses the idea that drug–target interactions should be considered as a means of inducing homeostatic responses within neurons, rather than solely focusing on the target (i.e., receptor–ligand) activity.

A substantially large number of molecular and cellular changes associated with ketamine are also associated with the physiological processes of sleep and wake [12]. While a general lack of consideration of sleep exists in both basic and clinical research involving ketamine’s antidepressant effects [11], some recent studies and hypotheses have begun to investigate the effects of ketamine from the perspective of the neurobiology of sleep and circadian rhythms. For example, a model by Duncan et al. [240] aims to uncover the effects of ketamine through the homeostatic and circadian components of sleep. In this context, treatment with ketamine leads not only to increased neuronal plasticity, slow-wave sleep (SWS), and enhanced sleep quality but also to modulation of circadian timing and output (Fig. 4c). These effects culminate in the reduction in the circadian mood component. Indeed, clinical studies have demonstrated an association between ketamine’s antidepressant effects and circadian rhythms [240, 241] as well as a correlation between ketamine-induced changes in the levels of BDNF, amount of SWS, quality of sleep, and subsequent mood changes in MDD [208, 242]. Moreover, it remains plausible that the antidepressant effects of ketamine are modulated by homeostatic sleep pressure and/or circadian time at the time of administration since cortical excitability in humans [243–245], and the anesthetic effects of ketamine in animal studies, are regulated by these factors [246, 247]. In any case, a significant translational gap exists due to the fact that most rodent species are nocturnal and sleep predominantly during the day when most drug treatments are given, whereas patients receive ketamine almost exclusively during the waking hours [11]. Understanding how sleep and circadian rhythms may influence treatment outcomes remains a very important theme for future studies in both basic and clinical research.

Building upon the themes of synaptic homeostasis and sleep, the hypothesis of encoding, consolidation, and renormalization in depression (ENCORE-D) proposes that ketamine’s rapid and sustained effects are produced and consolidated throughout wake and sleep in several phases [209] (Fig. 4d). It suggests that ketamine’s ability to increase cortical excitation triggers intrinsic mechanisms of synaptic plasticity both acutely and upon drug withdrawal, leading to the facilitated encoding of information and increase in synaptic strength. These changes are further consolidated during subsequent steps involving changes in transcription and protein synthesis. The final stage of ketamine-induced change is suggested to be reached on the night following treatment, during a period of deep sleep dominated by SWA. Here, ENCORE-D suggests that synapses in previously activated networks undergo renormalization of synaptic strength, a concept hypothesized within the framework of the the synaptic homeostasis hypothesis (SHY) [248, 249], ultimately giving rise to sustained alterations in network function. While remaining highly speculative and in need of experimental proof, the ENCORE-D framework provides several novel conceptual ideas about how the effects of rapid-acting antidepressants may be associated with the physiological mechanisms of wake and sleep in producing antidepressant outcomes [209].

## Conclusions

The past 50 years have transformed ketamine from a powerful battlefield anesthetic to the wonder drug of modern psychiatry, with new indications still being uncovered. Intensive research efforts aimed at understanding the precise mechanisms underlying ketamine’s effects have resulted in important advances in our understanding of depression and stimulated new concepts of molecular and cellular neuropharmacology. However, as with anything new, the glimmer of ketamine may have distracted both basic and clinical researchers from addressing fundamental issues. Certain questions, such as whether increasing doses in patients that respond poorly to lower doses is beneficial and whether anesthetic doses lack antidepressant effects, remain unanswered. There are also no comprehensive comparisons of the effectiveness of different administration routes or the rate and time administration. Moreover, the circadian time, dose, and method of administration in animal studies often differ significantly from those in clinical practice and lack proper translational validation. Future studies aimed at addressing these and other basic questions will hopefully advance our understanding of the pharmacological and neurobiological mechanisms of ketamine in the treatment of psychiatric disorders.

## Abbreviations

AMPAR: α-Amino-3-hydroxy-5-methyl-4-isoxazole-propionic acid receptor
BDNF: Brain-derived neurotrophic factor
BPRS: Brief Psychiatric Rating Scale
CADSS: Clinician-Administered Dissociative States Scale
DAT: Dopamine reuptake transporter
DHNK: Dehydronorketamine
DMN: Default-mode network
Drd1: Dopamine receptor D1
eEF2: Eukaryotic elongation factor 2
ECT: Electroconvulsive therapy
EEG: Electroencephalogram
ENCORE-D: Encoding, consolidation, and renormalization in depression
ERK: Extracellular signal-regulated kinase
FST: Forced swimming test
GABA: Gamma-aminobutyric acid
GSK3β: Glycogen synthase kinase 3 beta
HCN: Hyperpolarization-activated cyclic nucleotide
HDRS: Hamilton Depression Rating Scale
HMS: Hood’s Mysticism Scale
HNK: Hydroxynorketamine
IEG: Immediate early gene
IL-PFC: Infralimbic prefrontal cortex
im: Intramuscular
ip: Intraperitoneal
iv: Intravenous
LHb: Lateral habenula
LTP: Long-term potentiation
MADRS: Montgomery-Åsberg Rating Scale
MAPK: Mitogen-activated protein kinase
MDD: Major depressive disorder
MKP1: Mitogen-activated protein kinase phosphatase 1
mTOR: Mammalian target of rapamycin
NET: Noradrenaline reuptake transporter
NK: Norketamine
NMDAR: *N*-Methyl-d-aspartate receptor
PCC: Posterior cingulate cortex
PCP: Phencyclidine
PET: Positron emission tomography
PSD95: Postsynaptic density protein 95
p70-S6K: P70-S6 kinase
rTMS: Repetitive transcranial magnetic stimulation
sc: Subcutaneous
SERT: Serotonin reuptake transporter
sgACC: Subgenual anterior cingulate cortex
SWA: Slow-wave activity
SWS: Slow-wave sleep
TrkB: Tropomyosin receptor kinase B
VGSC: Voltage-gated sodium channel
